# Supplementary material for: Functional Prediction of Microbial Communities in Sediment Microbial Fuel Cells
Source: Bioengineering (Basel). 2023 Feb 3;10(2):199. doi: 10.3390/bioengineering10020199 (PMC9951962; doi:10.3390/bioengineering10020199)
Supplement: Supplementary file 1 [file bioengineering-10-00199-s001.zip › FigS3_posthoc_energy_re.pdf]

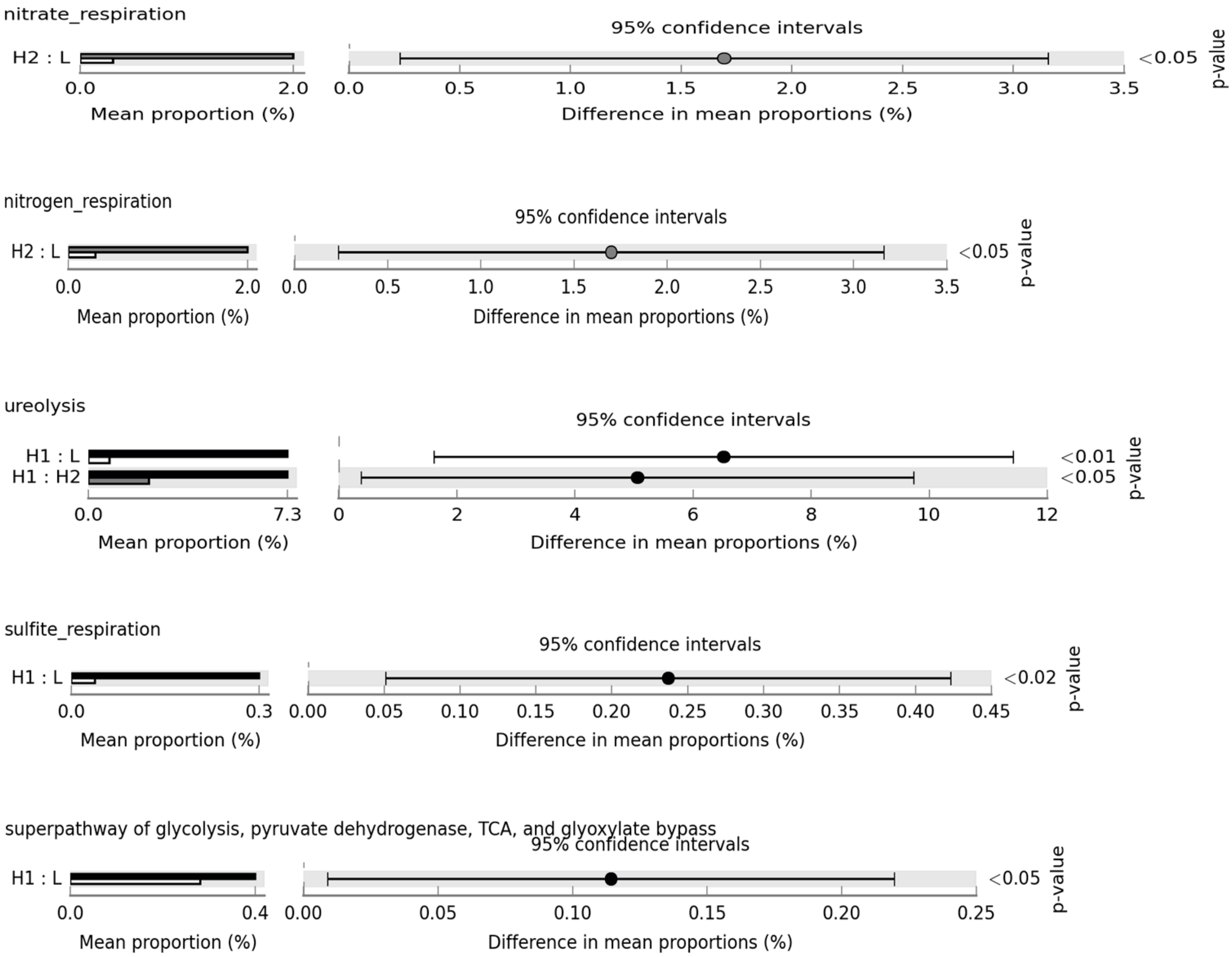

Figure S3. The significant post hoc assay by the Tukey–Kramer test for energy metabolism functional features.
