# Supplementary material for: Functional Prediction of Microbial Communities in Sediment Microbial Fuel Cells
Source: Bioengineering (Basel). 2023 Feb 3;10(2):199. doi: 10.3390/bioengineering10020199 (PMC9951962; doi:10.3390/bioengineering10020199)
Supplement: Supplementary file 1 [file bioengineering-10-00199-s001.zip › FigS4_posthoc_cofactor.pdf]

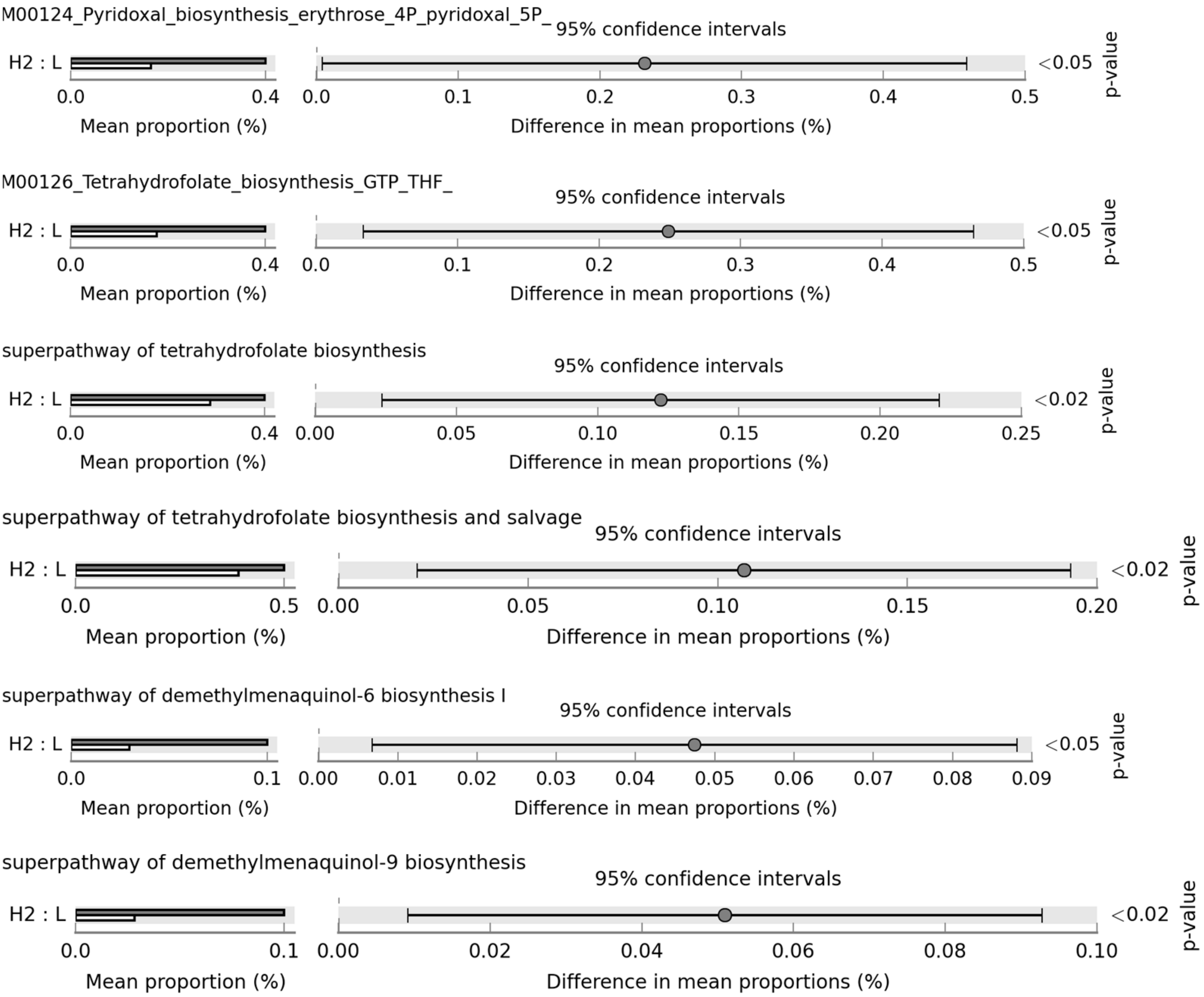

Figure S4. The significant post hoc assay by the Tukey–Kramer test for the functional features related to cofactor, carrier, and vitamin biosynthesis.
