# Supplementary material for: Functional Prediction of Microbial Communities in Sediment Microbial Fuel Cells
Source: Bioengineering (Basel). 2023 Feb 3;10(2):199. doi: 10.3390/bioengineering10020199 (PMC9951962; doi:10.3390/bioengineering10020199)
Supplement: Supplementary file 1 [file bioengineering-10-00199-s001.zip › Table s1 Shannon.pdf]

Table S1. Shannon diversity index of soil microbial community.

Samples	Shannon
D1	9.922
D2	8.445
D3	8.285
D4	9.883
D5	9.411
S1	9.215
S2	9.952
S3	10.237
S4	10.328
S5	9.836
D	10.991
DA	9.447
RF	11.021
RFA	8.57
Nr1	11.235
Nr2	11.248
Nr3	10.901
