# Supplementary material for: Functional Prediction of Microbial Communities in Sediment Microbial Fuel Cells
Source: Bioengineering (Basel). 2023 Feb 3;10(2):199. doi: 10.3390/bioengineering10020199 (PMC9951962; doi:10.3390/bioengineering10020199)
Supplement: Supplementary file 1 [file bioengineering-10-00199-s001.zip › TableS2_NSTI_FSU.pdf]

Table S2. Scores of the fraction of taxonomic units unused (FTU), the fraction of sequences unused (FSU), and the weighted NSTI

	Fraction of taxonomic units unused (FTU) (Tax4fun2 beta)	Fraction of sequences unused (FSU) (Tax4fun2 beta)	Weighted NSTI (PICRUStt2)
D1	0.83049	0.73743	0.10529451
D2	0.86337	0.8117	0.126038929
D3	0.84386	0.65774	0.082688743
D4	0.86321	0.7302	0.120133158
D5	0.85119	0.75681	0.104860133
S1	0.88913	0.89556	0.125323108
S2	0.87988	0.86613	0.181964255
S3	0.86602	0.80544	0.140493329
S4	0.84657	0.73201	0.135622074
S5	0.85861	0.79784	0.129562562
D	0.69096	0.61704	0.213360924
DA	0.66936	0.52232	0.188606015
RF	0.73346	0.72756	0.224126516
RFA	0.73227	0.85596	0.169761351
Nr1	0.94829	0.95161	0.515662881
Nr2	0.95033	0.95579	0.512702871
Nr3	0.91906	0.90397	0.404120219
