# Supplementary material for: Functional Prediction of Microbial Communities in Sediment Microbial Fuel Cells
Source: Bioengineering (Basel). 2023 Feb 3;10(2):199. doi: 10.3390/bioengineering10020199 (PMC9951962; doi:10.3390/bioengineering10020199)
Supplement: Supplementary file 1 [file bioengineering-10-00199-s001.zip › TableS3_Function_features.pdf]

Table S3. The functional features and the metabolic categories

Functional features	Metabolic pathways
**BugBase**	
M00019_Valine_isoleucine_biosynthesis_pyruvate_valine_2_oxobutanoate_isoleucine_	Amino acid metabolism
M00024_Phenylalanine_biosynthesis_chorismate_phenylalanine_	Amino acid metabolism
M00033_Ectoine_biosynthesis_	Amino acid metabolism
M00037_Melatonin_biosynthesis_tryptophan_serotonin_melatonin_	Amino acid metabolism
M00040_Tyrosine_biosynthesis_prephanate_pretyrosine_tyrosine_	Amino acid metabolism
M00042_Catecholamine_biosynthesis_tyrosine_dopamine_noradrenaline_adrenaline_	Amino acid metabolism
M00047_Creatine_pathway_	Amino acid metabolism
M00526_Lysine_biosynthesis_DAP_dehydrogenase_pathway_aspartate_lysine_	Amino acid metabolism
M00096_C5_isoprenoid_biosynthesis_non_mevalonate_pathway_	Biosynthesis of secondary metabolites
M00549_Nucleotide_sugar_biosynthesis_glucose_UDP_glucose_	Carbohydrate metabolism
M00162_Cytochrome_b6f_complex_	Energy metabolism
M00170_C4_dicarboxylic_acid_cycle_phosphoenolpyruvate_carboxykinase_type_	Energy metabolism
M00172_C4_dicarboxylic_acid_cycle_NADP_malic_enzyme_type_	Energy metabolism
M00347_Methanogenesis_formate_methane_	Energy metabolism
M00356_Methanogenesis_methanol_methane_	Energy metabolism
M00561_Methanogenesis_trimethylamine_methane_	Energy metabolism
M00562_Methanogenesis_dimethylamine_methane_	Energy metabolism
M00563_Methanogenesis_methylamine_methane_	Energy metabolism
M00567_Methanogenesis_CO2_methane_	Energy metabolism

M00186_Tungstate_transport_system_	Environmental Information Processing; Membrane transport
M00192_Putative_thiamine_transport_system_	Environmental Information Processing; Membrane transport
M00202_Oligogalacturonide_transport_system_	Environmental Information Processing; Membrane transport
M00213_L_Arabinose_transport_system_	Environmental Information Processing; Membrane transport
M00215_D_Xylose_transport_system_	Environmental Information Processing; Membrane transport
M00217_D_Allose_transport_system_	Environmental Information Processing; Membrane transport
M00225_Lysine_arginine_ornithine_transport_system_	Environmental Information Processing; Membrane transport
M00226_Histidine_transport_system_	Environmental Information Processing; Membrane transport
M00229_Arginine_transport_system_	Environmental Information Processing; Membrane transport
M00230_Glutamate_aspartate_transport_system_	Environmental Information Processing; Membrane transport
M00241_Vitamin_B12_transport_system_	Environmental Information Processing; Membrane transport
M00252_Lipooligosaccharide_transport_system_	Environmental Information Processing; Membrane transport
M00283_PTS_system_ascorbate_specific_II_component_	Environmental Information Processing; Membrane transport
M00306_PTS_system_fructose_specific_II_like_component_	Environmental Information Processing; Membrane transport
M00317_Manganese_iron_transport_system_	Environmental Information Processing; Membrane transport
M00324_Dipeptide_transport_system_	Environmental Information Processing; Membrane transport
M00335_Sec_secretion_system_	Environmental Information Processing; Membrane transport
M00429_Competence_related_DNA_transformation_transporter_	Environmental Information Processing; Membrane transport
M00446_RstB_RstA_two_component_regulatory_system_	Environmental Information Processing; Signal transduction
M00448_CssS_CssR_secretion_stress_response_two_component_regulatory_system_	Environmental Information Processing; Signal transduction
M00449_CreC_CreB_phosphate_regulation_two_component_regulatory_system_	Environmental Information Processing; Signal transduction
M00451_BasS_BasR_antimicrobial_peptide_resistance_two_component_regulatory_system_	Environmental Information Processing; Signal transduction
M00453_QseC_QseB_quorum_sensing_two_component_regulatory_system_	Environmental Information Processing; Signal transduction

M00456_ArcB_ArcA_anoxic_redox_control_two_component_regulatory_system_	Environmental Information Processing; Signal transduction
M00475_BarA_UvrY_central_carbon_metabolism_two_component_regulatory_system_	Environmental Information Processing; Signal transduction
M00476_ComP_ComA_competence_two_component_regulatory_system_	Environmental Information Processing; Signal transduction
M00479_DesK_DesR_membrane_lipid_fluidity_regulation_two_component_regulatory_system_	Environmental Information Processing; Signal transduction
M00483_NreB_NreC_dissimilatory_nitrate_nitrite_reduction_two_component_regulatory_system_	Environmental Information Processing; Signal transduction
M00484_YdfH_YdfI_two_component_regulatory_system_	Environmental Information Processing; Signal transduction
M00486_CitA_CitB_citrate_fermentation_two_component_regulatory_system_	Environmental Information Processing; Signal transduction
M00488_DcuS_DcuR_aerobic_C4_dicarboxylate_metabolism_two_component_regulatory_system	Environmental Information Processing; Signal transduction
M00498_NtrY_NtrX_nitrogen_regulation_two_component_regulatory_system_	Environmental Information Processing; Signal transduction
M00506_CheA_CheYBV_chemotaxis_two_component_regulatory_system_	Environmental Information Processing; Signal transduction
M00511_PleC_PleD_cell_fate_control_two_component_regulatory_system_	Environmental Information Processing; Signal transduction
M00513_LuxQN_CqsS_LuxU_LuxO_quorum_sensing_two_component_regulatory_system_	Environmental Information Processing; Signal transduction
M00515_FlrB_FlrC_polar_flagellar_synthesis_two_component_regulatory_system_	Environmental Information Processing; Signal transduction
M00342_Bacterial_proteasome_	Genetic Information Processing
M00343_Archaeal_proteasome_	Genetic Information Processing
M00360_Aminoacyl_tRNA_biosynthesis_prokaryotes_	Genetic Information Processing
M00060_Lipopolysaccharide_biosynthesis_KDO2_lipid_A_	Glycan metabolism, Lipopolysaccharide metabolism, carbohydrate metabolixm
M00107_Steroid_hormone_biosynthesis_cholesterol_prognenolone_progesterone_	Lipid metabolism
M00110_C19_C18_Steroid_hormone_biosynthesis_pregnenolone_androstenedione_estrone_	Lipid metabolism
M00361_Nucleotide_sugar_biosynthesis_eukaryotes_	Carbohydrate metabolism
M00124_Pyridoxal_biosynthesis_erythrose_4P_pyridoxal_5P_	Metabolism of cofactors and vitamins
M00126_Tetrahydrofolate_biosynthesis_GTP_THF_	Metabolism of cofactors and vitamins

M00048_Inosine_monophosphate_biosynthesis_PRPP_glutamine_IMP_	Nucleotide metabolism
M00313_indolepyruvate_ferredoxin_oxidoreductase	phenylalanine metabolism, tryptophan metabolism
M00418_Toluene_degradation_anaerobic_toluene_benzoyl_CoA_	Xenobiotics biodegradation
**FAPROTAX**	
aromatic_compound_degradation	Aromatic Compound Degradation
intracellular_parasites	Infectious disease
methanogenesis_by_CO2_reduction_with_H2	Energy metabolism
nitrate_reduction	Energy metabolism
nitrate_respiration	Energy metabolism
nitrogen_respiration	Energy metabolism
predatory_or_exoparasitic	Infectious disease
sulfite_respiration	Energy metabolism
ureolysis	Energy metabolism
**PICRUSt2**	
L-isoleucine biosynthesis I (from threonine)	Amino Acid Biosynthesis
L-lysine biosynthesis V	Amino Acid Biosynthesis
L-isoleucine biosynthesis II	Amino Acid Biosynthesis
L-isoleucine biosynthesis IV	Amino Acid Biosynthesis
superpathway of L-alanine biosynthesis	Amino Acid Biosynthesis
L-valine biosynthesis	Amino Acid Biosynthesis
catechol degradation to β-ketoadipate	Aromatic Compound Degradation
catechol degradation III (ortho-cleavage pathway)	Aromatic Compound Degradation

aromatic compounds degradation via β-ketoadipate	Aromatic Compound Degradation
superpathway of chorismate metabolism	Biosynthesis of the Aromatic Aminio Acids
starch biosynthesis	Carbohydrate Biosynthesis
UDP-2,3-diacetamido-2,3-dideoxy-α-D-mannuronate biosynthesis	Carbohydrate Biosynthesis
superpathway of UDP-N-acetylglucosamine-derived O-antigen building blocks biosynthesis	Carbohydrate Biosynthesis
sucrose degradation IV (sucrose phosphorylase)	Carbohydrate Degradation
glycogen degradation II (eukaryotic)	Carbohydrate Degradation
D-galacturonate degradation II	Carbohydrate Degradation
D-galactarate degradation I	Carboxylate Degradation
superpathway of D-glucarate and D-galactarate degradation	Carboxylate Degradation
2-methylcitrate cycle II	Carboxylate Degradation
superpathway of tetrahydrofolate biosynthesis and salvage	Cofactor, Carrier, and Vitamin Biosynthesis
superpathway of menaquinol-9 biosynthesis	Cofactor, Carrier, and Vitamin Biosynthesis
superpathway of menaquinol-6 biosynthesis I	Cofactor, Carrier, and Vitamin Biosynthesis
superpathway of demethylmenaquinol-6 biosynthesis I	Cofactor, Carrier, and Vitamin Biosynthesis
superpathway of demethylmenaquinol-9 biosynthesis	Cofactor, Carrier, and Vitamin Biosynthesis
superpathway of menaquinol-10 biosynthesis	Cofactor, Carrier, and Vitamin Biosynthesis
superpathway of heme biosynthesis from glycine	Cofactor, Carrier, and Vitamin Biosynthesis
superpathway of tetrahydrofolate biosynthesis	Cofactor, Carrier, and Vitamin Biosynthesis
pyridoxal 5'-phosphate biosynthesis I	Cofactor, Carrier, and Vitamin Biosynthesis
superpathway of fatty acid biosynthesis initiation (E. coli)	Fatty Acid and Lipid Biosynthesis
archaetidylserine and archaetidylethanolamine biosynthesis	Fatty Acid and Lipid Biosynthesis
superpathway of glycolysis, pyruvate dehydrogenase, TCA, and glyoxylate bypass	Generation of Precursor Metabolites and Energy

Bifidobacterium shunt	Generation of Precursor Metabolites and Energy
nitrifier denitrification	Generation of Precursor Metabolites and Energy
superpathway of glyoxylate bypass and TCA	Generation of Precursor Metabolites and Energy
pyrimidine deoxyribonucleotides de novo biosynthesis III	Nucleoside and Nucleotide Biosynthesis
enterobactin biosynthesis	Secondary Metabolite Biosynthesis
isoprene biosynthesis II (engineered)	Secondary Metabolite Biosynthesis
**Tax4Fun2**	
Regulation of actin cytoskeleton	Cellular Processes
Oocyte meiosis	Cellular Processes
Cellular senescence	Cellular Processes
Meiosis - yeast	Cellular Processes
Focal adhesion	Cellular Processes
Hippo signaling pathway	Environmental Information Processing, Signal transduction
Wnt signaling pathway	Environmental Information Processing, Signal transduction
Ras signaling pathway	Environmental Information Processing, Signal transduction
AMPK signaling pathway	Environmental Information Processing, Signal transduction
Sphingolipid signaling pathway	Environmental Information Processing, Signal transduction
cAMP signaling pathway	Environmental Information Processing, Signal transduction
Chronic myeloid leukemia	Human Diseases
Amyotrophic lateral sclerosis (ALS)	Human Diseases
Salmonella infection	Human Diseases, Infectious disease
Shigellosis	Human Diseases, Infectious disease
Biotin metabolism	Metabolism of cofactors and vitamins

Glycosaminoglycan degradation	Metabolism, Glycan biosynthesis and metabolism
Fatty acid biosynthesis	Metabolism, Lipid
Glycerophospholipid metabolism	Metabolism, Lipid
Indole alkaloid biosynthesis	Metabolism, Biosynthesis of other secondary metabolites
Methane metabolism	Metabolism, Energy
Steroid degradation	Metabolism, Xenobiotics biodegradation and metabolism
Isoquinoline alkaloid biosynthesis	Metabolism, Biosynthesis of other secondary metabolites
Furfural degradation	Metabolism, Xenobiotics biodegradation and metabolism
Long-term potentiation	Organismal Systems
Vascular smooth muscle contraction	Organismal Systems
Inflammatory mediator regulation of TRP channels	Organismal Systems
Synaptic vesicle cycle	Organismal Systems
Platelet activation	Organismal Systems
Protein digestion and absorption	Organismal Systems
GnRH signaling pathway	Organismal Systems
Fc gamma R-mediated phagocytosis	Organismal Systems
Glucagon signaling pathway	Organismal Systems
Carbohydrate digestion and absorption	Organismal Systems
Cholinergic synapse	Organismal Systems
Renin secretion	Organismal Systems
Fc epsilon RI signaling pathway	Organismal Systems
Longevity regulating pathway - multiple species	Organismal Systems
