# Supplementary material for: Functional Prediction of Microbial Communities in Sediment Microbial Fuel Cells
Source: Bioengineering (Basel). 2023 Feb 3;10(2):199. doi: 10.3390/bioengineering10020199 (PMC9951962; doi:10.3390/bioengineering10020199)
Supplement: Supplementary file 1 [file bioengineering-10-00199-s001.zip › Table_S4.pdf]

**Table S4.** The taxonomy assignment of the functional features related to energy metabolism predicted by FAPROTAX

| Functional features | Bacteria |
|---|---|
| nitrate_reduction (38 records) | *g__Bilophila;__* |
| | *g__Desulfovibrio;s__Desulfovibrio_oxamicus* |
| | *s__Geobacter_argillaceus* |
| | *s__Bacillus_funiculus* |
| | *g__Anaerostipes;__* |
| | *g__Veillonella;__* |
| | *s__Elioraea_tepidiphila* |
| | *s__Pedomicrobium_americanum* |
| | *g__Ensifer;__* |
| | *g__Rhodoplanes;__* |
| | *g__Gemmobacter;__* |
| | *g__Paracoccus;__* |
| | *g__Magnetospirillum;__* |
| | *g__Aeromonas;__* |
| | *g__Shewanella;__* |
| | *s__Shewanella_baltica* |
| | *g__Achromobacter;__* |
| | *g__Cupriavidus;__* |
| | *s__Cupriavidus_oxalaticus* |
| | *g__Aquabacterium;__* |
| | *g__Ideonella;__* |
| | *g__Azoarcus;__* |
| | *s__Azoarcus_tolulyticus* |
| | *g__Azospira;__* |
| | *g__Azospira;s__uncultured_Rhodocyclaceae* |
| | *g__Dechloromonas;__* |
| | *g__Thauera;__* |
| | *g__Zoogloea;__* |
| | *g__Sutterella;__* |
| | *g__Sutterella;s__gut_metagenome* |
| | *s__Sutterella_wadsworthensis* |
| | *g__Sutterella;s__uncultured_bacterium* |
| | *g__Enterobacter;__* |
| | *g__Klebsiella;__* |
| | *g__Haemophilus;__* |

| | |
|---|---|
| | *g__Vibrio;__*<br>*g__Stenotrophomonas;__*<br>*g__Opitutus;__* |
| nitrate_respiration (19) | *g__Bilophila;__*<br>*s__Desulfovibrio_oxamicus*<br>*s__Geobacter_argillaceus*<br>*g__Rhodoplanes;__*<br>*g__Gemmobacter;__*<br>*g__Paracoccus;__*<br>*g__Magnetospirillum;__*<br>*g__Shewanella;__*<br>*s__Shewanella_baltica*<br>*g__Achromobacter;__*<br>*g__Aquabacterium;__*<br>*g__Azoarcus;__*<br>*s__Azoarcus_tolulyticus*<br>*g__Azospira;__*<br>*g__Azospira;s__uncultured_Rhodocyclaceae*<br>*g__Dechloromonas;__*<br>*g__Thauera;__*<br>*g__Zoogloea;__*<br>*g__Stenotrophomonas;__* |
| nitrogen_respiration (20) | *g__Bilophila;__*<br>*s__Desulfovibrio_oxamicus*<br>*s__Geobacter_argillaceus*<br>*s__anammox_bacterium*<br>*g__Rhodoplanes;__*<br>*g__Gemmobacter;__*<br>*g__Paracoccus;__*<br>*g__Magnetospirillum;__*<br>*g__Shewanella;__*<br>*s__Shewanella_baltica*<br>*g__Achromobacter;__*<br>*g__Aquabacterium;__*<br>*g__Azoarcus;__*<br>*s__Azoarcus_tolulyticus*<br>*g__Azospira;__*<br>*g__Azospira;s__uncultured_Rhodocyclaceae* |

| | |
|---|---|
| | *g__Dechloromonas;__* |
| | *g__Thauera;__* |
| | *g__Zoogloea;__* |
| | *g__Stenotrophomonas;__* |
| ureolysis (12) | *s__Bacillus_funiculus* |
| | *g__Roseomonas;__* |
| | *s__Roseomonas_lacus* |
| | *s__Roseomonas_rubra* |
| | *g__Azospirillum;__* |
| | *s__Azospirillum_sp.* |
| | *g__Mesorhizobium;__* |
| | *s__Zavarzinia_compransoris* |
| | *g__Methylophilus;__* |
| | *s__Nitrosomonas_nitrosa* |
| | *g__Herbaspirillum;__* |
| | *g__Azotobacter;__* |
| sulfite_respiration (2) | *g__Desulfobulbus;__* |
| | *s__uncultured_Desulfobulbus* |
| methanogenesis_by_CO2_reduction_with_H2 (13) | *g__Methanobacterium* |
| | *g__Methanobacterium;s__uncultured_bacterium* |
| | *o__Methanomicrobiales;__;__;__* |
| | *s__uncultured_Methanomicrobiales* |
| | *g__Methanolinea;__* |
| | *g__Methanolinea;s__uncultured_archaeon* |
| | *s__uncultured_Methanomicrobiales* |
| | *g__Methanoregula;__* |
| | *g__Methanoregula;s__uncultured_methanogenic* |
| | *g__Methanospirillum;__* |
| | *s__Methanospirillum_psychrodurum* |
| | *g__Methanospirillum;s__uncultured_archaeon* |
| | *g__Methanosarcina;__* |
