# Supplementary figures and images for: Functional Prediction of Microbial Communities in Sediment Microbial Fuel Cells
Source: Bioengineering (Basel). 2023 Feb 3;10(2):199. doi: 10.3390/bioengineering10020199 (PMC9951962; doi:10.3390/bioengineering10020199)

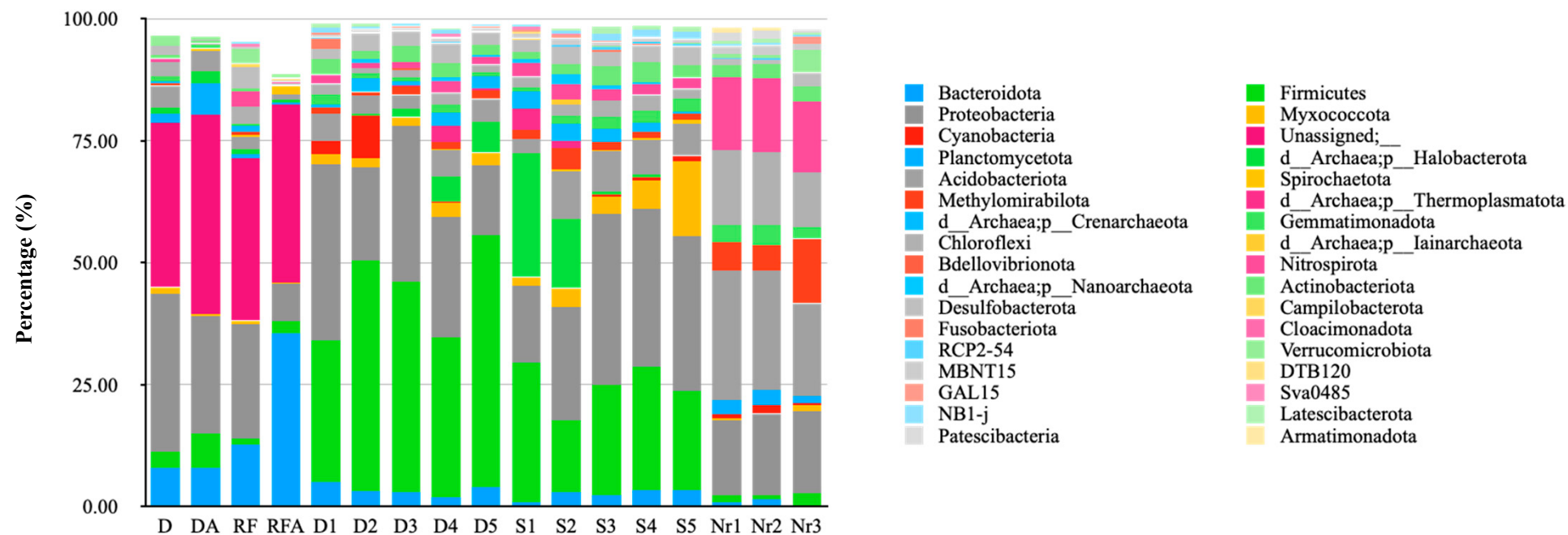

**Figure S1.** Relative abundance of phyla of the soil microbial communities.

Supplement: Supplementary file 1 [file bioengineering-10-00199-s001.zip › Fig s1 relative .pdf]
